# Patient characteristics and cardiac surgical outcomes at a tertiary care hospital in Kenya, 2008–2017: a retrospective study

**DOI:** 10.7717/peerj.11191

**Published:** 2021-05-10

**Authors:** Tamara Chavez-Lindell, Bob Kikwe, Anthony Gikonyo, Agricola Odoi

**Affiliations:** 1 Department of Biomedical and Diagnostic Sciences, University of Tennessee, Knoxville, TN, United States of America; 2 The Karen Hospital, Nairobi, Kenya

**Keywords:** Patient characteristics, Open heart surgery, Kenya, Cardiothoracic surgical outcomes, Firth logistic regression model, Cardiac surgery, 30-day mortality, In-hospital mortality, Intra-surgical mortality, Out-of-hospital mortality

## Abstract

**Background:**

Cardiac surgeries are high risk procedures that require specialized care and access to these procedures is often limited in resource-poor countries. Although fatalities for surgical patients across Africa are twice that of the global rate, cardiac surgical mortality continent-wide is only slightly higher than all-surgical mortality. Understanding demographic and health characteristics of patients and the associations of these characteristics with morbidity and mortality events is important in guiding care decisions. Therefore, the objectives of this study were to: (a) describe the characteristics of cardiac surgical patients; (b) identify the associations between these characteristics and morbidity and mortality events following cardiac surgery.

**Methods:**

Patient characteristics and post-surgical complications were abstracted for all cardiac surgical patients treated at a tertiary care hospital in Kenya from 2008 to 2017. Descriptive analyses of demographic factors, co-morbidities, peri-operative conditions, and post-surgical complications were conducted for adult and pediatric patients. Cochran-Armitage trend test was used to assess temporal trends in risk of death. Multivariable ordinary logistic and Firth logistic models were used to investigate predictors of surgical outcomes.

**Results:**

The study included a total of 181 patients (150 adult and 31 pediatric patients). Most (91.3%) adult patients had acquired conditions while 45.2% of the pediatric patients had congenital defects. Adult patients tended to have co-morbid conditions including hypertension (16.7%), diabetes mellitus (7.3%), and nephropathy (6.7%). Most patients (76.0% adults and 96.8% pediatric patients) underwent ≤ 2 surgical procedures during their hospital stay. Seventy percent of adult and 54.8% of the pediatric patients experienced at least one post-surgical complication including mediastinal hemorrhage, acute kidney injury and death. Patient characteristics played the greatest roles in predicting post-surgical complications. For adult patients, significant predictors of acute kidney injury included atrial fibrillation (OR = 18.25; *p* = .001), mitral valve replacement (OR = 0.14; *p* = .019), and use of cardiopulmonary bypass (OR = 0.06; *p* = .002). Significant predictors of 30-day mortality were age (OR = 1.05; *p* = .015) and atrial fibrillation (OR = 4.12, *p* = .018). Although the number of surgeries increased over the decade-long study period, there were no significant (*p* = .467) temporal trends in the risk of death.

**Conclusions:**

Awareness of demographic and peri-surgical factors that are predictors of complications is useful in guiding clinical decisions to reduce morbidity and mortality. Identification of co-morbidities as the most useful predictors of post-surgical complications suggests that patient characteristics may be a larger contributor to the incidence of complications than surgical practices.

## Introduction

Cardiac surgical procedures require specialized care which tends to be more readily available in developed countries than low to middle income countries (LMIC). This is generally a result of shortage, in LMIC, of both the necessary medical specialists and the medical infrastructure required for these procedures. Yet there is evidence of increasing need for cardiac surgical care (Zilla et al., 2018). This makes it necessary for some patients to travel abroad to access the specialized care they need. Unfortunately, only a small proportion of the population can afford to do this. The opening, in recent years, of a number of tertiary care centers that provide a wide range of outpatient services, as well as surgical care in the areas of orthopedics, gastroenterology, oncology, obstetrics, pediatrics, and cosmetic surgery, has improved availability of these specialized medical services and reduced wait times and expense, compared to traveling abroad for care.

Intra-continental medical travel has increased rapidly over the past two decades. Despite good estimation of the increase in medical tourism worldwide, there is limited understanding of its impact on healthcare provision and health outcomes in Africa (Mogaka et al., 2017). Although some reports indicate that the mortality rate for all surgical patients in Africa is twice the global mortality rate (Biccard et al., 2018), it may be argued that lack of access to surgical procedures is an equally important problem. Yet elaboration of risk estimates associated with surgical procedures in resource-constrained settings is limited. In the absence of conditional risk estimates, prospective patients and medical providers cannot make personalized risk-benefit assessments and decisions. Drawing from patients receiving care in 25 African countries, the African Surgical Outcomes Study (ASOS) Group created a tool that may be used in preparation for elective surgery to identify high-risk patients and optimize their post-operative observation (Kluyts et al., 2018). The application of such a surgical risk calculator is anticipated to reduce surgery-associated mortality across the continent, if patients identified as high-risk elect to forego surgery. However, the surgical risk calculator does not provide specific risk estimates for cardiac procedures. Additionally, given the baseline cause-specific mortality for cardiac patients and the quality of life impacts that many cardiac patients experience as a result of their condition, cardiac surgical procedures are often viewed as less elective than essential. Few studies have assessed the post-surgical outcomes of such procedures at tertiary facilities in LMIC.

Since overall surgical outcomes may be significantly impacted by resource limitations extraneous to quality of care, reducing complications is an important goal. Given the rapidly expanding availability of specialized care in LMIC, accurate identification of patients at greatest risk of post-surgical complications is critical. With that in mind, the objectives of this study were to: (a) describe the characteristics of cardiac surgical patients, and (b) identify the associations between these characteristics and morbidity and mortality events following cardiac surgery at a tertiary hospital in Kenya between 2008 and 2017.

## Material and Methods

### Study design and data source

This is a retrospective study that used secondary data extracted from patient records obtained from a tertiary hospital in Kenya. Retrospective secondary data of all (*n* = 181) cardiac surgical patients who received care at the tertiary hospital from 2008 through 2017 were abstracted and included in the study. The inclusion criteria were patients on whom a standard median sternotomy had been performed for repair or correction of cardiac defects. The exclusion criteria were patients who underwent surgical procedures for abdominal aortic aneurysm, patent ductus arteriosus (PDA) closure and pericardiotomy.

### Data analysis

#### Descriptive analyses

Descriptive statistical analyses and assessments of univariable associations were performed in SPSS (IBM Corp. Released 2016. IBM SPSS Statistics for Windows, Version 24.0. Armonk, NY: IBM Corp.). These analyses were conducted separately for adult and pediatric patients. Analyses included computation of frequencies of demographic factors, co-morbidities, peri-operative conditions, and post-surgical complications. Simple associations between surgical complications and potential pre- and peri-operative predictors of surgical outcomes were assessed using X^2^ or Fisher’s exact tests, as appropriate. Cochran-Armitage trend test was used to assess temporal trends in risk of death. Most of the variables assessed were categorical. The few continuous variables assessed were age, weight, height, calculated body mass index (BMI), creatinine and creatinine clearance. Shapiro–Wilk test was used to assess normality of distribution of age and BMI for each age category (adult and pediatric). Means were computed for normally distributed variables while medians and interquartile ranges (IQR) were computed for those that were not normally distributed. Age and BMI were also categorized into age groups (<10, 10–17, 18–34, 35–49, 50–64, and ≥65 years) and BMI groupings [<5th percentile (Underweight), 5th-85th percentile (Normal or Healthy Weight), 85th-95th percentile (Overweight), and >95th percentile (Obese)] to enhance interpretation of results.

The patients had a wide range of cardiac defects which were classified as congenital defects only, acquired defects only, or both congenital and acquired defects. The total number of defects per patient was also calculated. In addition to the summary of defects, a composite score reflecting the clinical complexity of each patient’s condition was computed by assigning one point for each of the following pre- or peri-operative factors identified in the patient record: age greater than or equal to 50 years, pulmonary arterial pressure >55 mmHg, atrial fibrillation, left atrial thrombus, infective endocarditis, presence of heart block, peripheral arterial disease, chronic obstructive pulmonary disease, lower respiratory tract infection, hypertension, diabetes mellitus, nephropathy, seizure disorder, cerebrovascular accident, Human Immunodeficiency Virus (HIV), thyroid derangement (defined as any diagnosed thyroid excess or insufficiency and consequent treatment in a patient, including anti-thyroid medication or thyroid hormone replacement), atrial septal defect, ventricular septal defect, tetralogy of Fallot, atrioventricular septal defect, Ebstein anomaly, bicuspid aortic valve defect, pulmonary valve stenosis, total anomalous pulmonary veins, mitral cleft, subaortic ridge, mitral valve regurgitation, mitral valve stenosis, aortic regurgitation, aortic stenosis, tricuspid regurgitation, ascending aortic aneurysm, sinus of Valsalva aneurysm, 3 graft Coronary Artery Bypass Graft (CABG) procedure, 4 graft CABG procedure, and duration of procedure >4 h. The median and interquartile ranges of the number of procedures per patient were also computed. The number of procedures per patient was defined as the number of procedures performed on a single patient during a single hospital stay.

Patients were assessed for a range of complications, including: renal impairment, pneumothorax, pneumonia, acute kidney injury, mediastinal hemorrhage, pericardial effusion, sepsis, stroke, re-operation (sternotomy), cardiogenic shock, atrial fibrillation (new onset), heart failure (new onset), and death. All deaths observed in this study occurred within 30 days following the surgery. Therefore, assessment of deaths included 30-day mortality, defined as death within 30 days of the surgical operation (Edmunds et al., 1996; Akins et al., 2008). The overall 30-day mortality was further sub-classified as: (1) intra-surgical mortality, defined as death occurring during the surgical operation; (2) in-hospital mortality, defined as death within 30 days of the surgical operation while the patient was still hospitalized; and (3) out-of-hospital mortality, defined as death occurring within 30 days of the surgical operation but after discharge from the hospital. Patient characteristics and the computed composite scores were assessed for univariable associations with post-surgical complications using X^2^or Fisher’s Exact tests, as appropriate.

### Investigation of predictors of adverse surgical outcomes

Multivariable logistic regression models were fit to the data to assess the association between the pre-/peri-operative characteristics and selected severe intra- and post-surgical complications, including pneumonia, acute kidney injury, cardiogenic shock, sepsis, stroke, and both overall 30-day mortality and intra-surgical mortality. Model building was done in SAS (SAS Institute. Released 2013. SAS Statistics for Windows, Version 9.4. Cary, NC: SAS Institute Inc.), using a backwards stepwise approach that allowed assessment for potential confounders. Variables were excluded from the model at *p* > 0.05. When removal of a variable resulted in a change of ≥20% of the coefficients of any of the variables still in the model, the removed variable was considered a confounder and manually included in the model, regardless of significance level. In a number of the models, age was included as a confounder. Due to the small number of complications occurring among pediatric patients and the biological differences between pediatric and adult patients, the former cases were excluded from the multivariable model building process. Therefore, the models were only fit for adult patients. Given the small number of complications recorded, Firth bias corrected logistic models, which are more appropriate for modeling rare events, were used. Goodness-of-fit of the models were assessed using the Hosmer-Lemeshow test.

#### Ethical approval

Ethical approvals for this study were granted by both the TKH Bioethics and Research Committee, Nairobi, Kenya (Number: TKH/ADMIN/CEO/18/12/17) and the University of Tennessee Institutional Review Board, Knoxville, TN, USA (Number: 18-04738-XM). Since this was a retrospective study that used secondary data which did not identify patients and did not involve direct participation of patients, consent was not required.

## Results

### Descriptive statistics and univariable associations

Patients included in this study ranged in age from 0 to 83 years. Age was normally distributed for the whole dataset (Shapiro–Wilk test: *p* = .088) and pediatric patients (Shapiro–Wilk test: *p* = .076) but non-normally distributed for adult patients (Shapiro–Wilk test: *p* = .003). Adults had a median age of 40 years (Interquartile range [IQR]: 32, 54) while the median age of pediatric patients was 10 years (IQR: 6, 14). Significantly (*p* = .002) more adult females (62.7%) than adult males (37.3%) underwent cardiac surgery (Table 1). However, there were no significant gender differences among children (54.8% females vs 45.2% males, *p* = .593) (Table 1). Given the differences between adult and pediatric populations with regard to their disease etiology and surgical outcome frequencies, the analyses were performed separately for the two age categories.

**Table 1:** Selected demographic characteristics and co-morbidities of cardiac surgical patients seen at a tertiary Hospital in Kenya, 2008–2017.

**Patient factor**	**% Adult patients** **(*n* = 150)**	**% Pediatric patients** **(*n* = 31)**
**Gender**		
Male	37.3	45.2
Female	62.7	54.8
**Number of complications**		
0	30.0	45.2
1	43.3	35.5
2 to 3	19.4	16.1
4 to 6	7.3	0.0
7 to 10	0.0	3.2
**Substance use**		
Alcohol	22.7	3.2
Smoking	9.3	3.2
Khat	2.7	0.0
Other	0.0	0.0
**BMI status**		
Underweight	10.7	87.1
Healthy weight	50.0	12.9
Overweight/Obese	39.3	0.0
**Co-morbidities**		
Human Immunodeficiency Virus (HIV)	3.4	0.0
Diabetes mellitus	7.3	0.0
Hypertension	16.7	6.5
Nephropathy	6.7	0.0
Thyroid derangement	4.0	3.2
**Number of defects**		
1 or 2	55.4	74.2
3 to 6	44.6	25.8
**Type of defects**		
Congenital	5.3	45.2
Acquired	91.3	41.9
Both congenital and acquired	3.3	12.9
**Number of surgical procedures during stay**		
0 to 2	76.0	96.8
3 to 5	24.0	3.2

Overall, post-surgical complications occurred in 70.0% of adult cardiac surgical cases and 54.8% of pediatric cardiac surgical cases (Table 1). However, a large proportion of patients in each age category experienced just one complication with 73.3% of adult patients and 80.7% of the pediatric patients having one or no post-surgical complications (Table 1). Complications among adult patients ranged from 0 to 6, with a median of 1 (IQR: 0, 2). Conversely, complications were much less frequent among pediatric patients, but had a wider distribution (range: 0–10; median = 1, IQR: 0, 1). It is worth noting that only one pediatric patient had 10 complications while the rest of the pediatric patients had 0 to 3 complications.

The most common post-surgical complication among adults was pleural effusion, which occurred in 33.3% of the patients. However, the condition is not uncommon among cardiac surgical patients and did not represent a finding of concern. The most commonly identified serious complication among adults was mediastinal hemorrhage, which occurred in 30.7% of the adult patients. Other serious complications among adults included sepsis (16.7%) and pericardial effusion (14.0%). Mediastinal hemorrhage was also the most frequent complication among pediatric cases, occurring in 38.7% of these patients. Other pediatric complications included acute kidney injury (12.9%) and pneumothorax (9.7%). New diagnoses of atrial fibrillation were relatively infrequent (13.3%) among adult patients and no new diagnoses occurred among the pediatric cases.

Overall, a small proportion of patients reported substance use (Table 1). Based on univariable (simple) associations, among adults, use of alcohol had no significant (*p* > .05) association with any post-surgical complications. Smoking was not significantly (*p* > .05) associated with any post-surgical complications, with the exception of development of psychosis (*p* = .003). Reported pediatric smoking and alcohol use were extremely low and substance use was not significantly (*p* > .05) associated with any post-surgical complications among pediatric patients. The majority (87.1%) of the pediatric patients were underweight and none were overweight/obese. Conversely, 39.3% of adult patients were overweight/obese while 10.7% were underweight (Table 1).

All deaths that occurred were from cardiac causes and occurred among 10.0% of adult and 6.5% of pediatric patients. No significant (*p* = .538) difference in the proportion of deaths among the two age classes was observed. Ages of adult patients that died were normally distributed with a mean of 51.8 years (standard deviation [s.d.] = 15.1 years). Pediatric deaths (*n* = 2) occurred in both the <10 and 10–17-year age groups. In-hospital mortality was more common than out-of-hospital mortality and comprised 73.3% of adult deaths and 100% of pediatric deaths. Intra-surgical mortality was responsible for 20.0% of the adult deaths, while out-of-hospital death occurred in just one adult patient (6.7%). Post-surgical deaths among adults occurred at a median of 7 days (IQR: 5, 11), while both pediatric deaths occurred at 5 days post-surgery. All 30-day mortality events were due to cardiac causes.

The proportion of patients with co-morbid conditions was generally low among both pediatric and adult patients (Table 2). Among adult patients, the most frequent co-morbidity was overweight/obesity (BMI >85th percentile), which accounted for 39.3% of the cases. The BMI >85th percentile was significantly (*p* = .022) associated with 30-day mortality. Additional co-morbidities that had significant unadjusted (univariable) association with 30-day post-surgical mortality among adults were pre-existing atrial fibrillation (38.0%, *p* = .016) and Heart Failure with Reduced Ejection Fraction (HFREF) (24.7%, *p* = .030) (Table 2). Pediatric patients had a much lower proportion of co-morbidities, encompassing a maximum of 12.9%, for HFREF. However, HFREF was not significantly (*p* = .106) associated with 30-day mortality for these patients (Table 2). The only variable that had significant (*p* = .010) unadjusted (univariable) association with 30-day mortality among pediatric patients was hypertension, which occurred in 6.5% of these patients (Table 2). Optimization of heart failure was identified pre-operatively in the majority of both adult (86.7%) and pediatric (64.5%) patients. However, this was not significantly associated with 30-day mortality in adults (*p* = .139) or pediatric patients (*p* = .657).

**Table 2:** Selected co-morbidities and their unadjusted (univariable) associations with 30-day mortality among cardiac surgical patients seen at a tertiary hospital in Kenya, 2008–2017.

**Co-morbidity**	**% Adult Cases** **(*n* = 150)**	*p*-value^a^	**% Pediatric Cases** **(*n* = 28)**	*p*-value^a^
BMI >85th percentile (overweight/obesity)	39.3	**.022**	0.0	–
Atrial fibrillation	38.0	**.016**	6.5	.701
Heart failure with reduced ejection fraction	24.0	**.030**	12.9	.106
Hypertension	16.7	..273	6.5	**.010**
Chronic obstructive pulmonary disease	10.0	.174	6.5	.701
Diabetes	7.3	.251	0.0	–
Nephropathy	6.7	1.000	0.0	–
Lower respiratory tract infection	4.0	.579	0.0	–
Human immunodeficiency virus	3.3	.447	0.0	–
Peripheral vascular disease	3.3	.448	0.0	–

**Notes.**

a Based on Chi-square test.

As might be expected, defects among adult patients largely represented acquired conditions (91.3%), the most frequent of which was mitral stenosis, identified in 57.3% of adult patients (Table 3). Other frequent conditions among adults included mitral regurgitation (40.7%), tricuspid regurgitation (33.3%), and aortic regurgitation (24.0%) (Table 3). The distribution of specific defects varied by age category but was only significantly different for mitral regurgitation (*p* = .003) and mitral stenosis (*p* = .005). Both defects were most common among the 18–34-year age group, with this age group making up 42.6% and 40.7% of all mitral regurgitation and mitral stenosis cases, respectively. The number of defects in adult patients ranged from 0 to 6, with a median of 1 (IQR: 2, 3).

**Table 3:** Selected cardiac defect types and their association with 30-day mortality among cardiac surgical patients seen at a tertiary hospital in Kenya, 2008–2017.

**Defect type**	**% Adult patients** **(*n* = 150)**	*p*-value^a^	**% Pediatric patients** **(*n* = 28)**	*p*-value^a^
**Congenital cardiac defects**	**5.3**		**45.2**	
Atrial septal	6.7	1.000	16.1	.521
Atrioventricular septal	0	–	12.9	.574
Ebstein anomaly	0	–	3.2	.790
Mitral cleft	0.7	.738	3.2	.790
Subaortic ridge	0	–	6.5	.701
Tetralogy of fallot	0	–	9.7	.632
Sinus of valsalva aneurysm	0.7	.738	0	–
Ventricular septal defect	1.3	.635	22.6	.338
**Acquired cardiac defects**	**91.3**		**41.9**	
Ascending aortic aneurysm	2.7	.499	3.2	.790
Aortic regurgitation	24.0	.702	16.1	.178
Aortic stenosis	12.7	.935	0	–
Bicuspid aortic valve	0.7	.738	6.5	**.010**
Coronary artery disease – double-vessel disease	1.3	.058	0	–
Coronary artery disease – left main disease	2.0	.176	0	–
Coronary artery disease – single-vessel disease	3.3	.448	0	–
Coronary artery disease – triple-vessel disease	7.3	.917	0	–
Mitral regurgitation	40.7	.618	35.5	.657
Mitral stenosis	57.3	.741	19.4	.474
Pulmonary valve stenosis	0	–	6.5	.701
Tricuspid regurgitation	33.3	.248	25.8	.389
**Both congenital & acquired**	**3.3**		**12.9**	

**Notes.**

a Based on Chi-square test.

The most frequently identified defects among pediatric patients was mitral regurgitation (35.5%), followed by tricuspid regurgitation (25.8%), and ventricular septal defect (22.6%). However, as the proportions of specific defect types suggest, the clinical presentation among pediatric cases varied significantly (*p* = .008) by age. For instance, whereas children less than 10 years of age had mainly congenital defects (66.7%), those 10-17 years of age had mainly acquired defects (68.8%). The number of conditions identified in pediatric patients ranged from 0 to 6, with a median of 2 (IQR: 1, 3).

Most of the defects were not significantly associated with death or other severe post-surgical complications, either for adults or children (Table 3). The few exceptions of significant unadjusted (univariable) associations include: bicuspid aortic valve defect, which had significant unadjusted (univariable) associations with both 30-day mortality (*p* = .010) and sepsis (*p* = .010) among the pediatric patients; aortic regurgitation, which had a significant (*p* = .020) unadjusted (univariable) association with cardiogenic shock again among pediatric patients; mitral cleft, which had a significant (*p* = .025) unadjusted association with sepsis among adult patients; mitral stenosis, which had a significant (*p* = .048) univariable association with acute kidney injury among adults; triple vessel disease, which had a significant (*p* = .002) univariable association with acute kidney injury among adults; left main coronary artery disease, which had a significant (*p* = .004) univariable association with cardiogenic shock among adults; and sinus of Valsalva aneurysm, which had a significant (*p* = .025) univariable association with sepsis among adults.

Since the specified defect-outcome associations were inconsistent across age categories, a “clinical complexity score” was calculated to provide a single measure of underlying health risk for each patient that could be compared to outcomes. The resulting patient scores, calculated from the presence of 37 distinct demographic, defect, and co-morbidity factors, ranged from 0–10 (median =4, IQR: 2, 5). Pediatric patients had a median clinical complexity score of 3 (IQR: 2, 4), while adults had a median score of 4 (IQR: 3, 5). The clinical complexity score was compared to selected severe post-surgical outcomes, with the following findings: among adults, a greater than the median score had significant univariable associations with overall 30-day mortality (*p* = .014), acute kidney injury (*p* = .002) and heart failure (*p* < .001). No significant univariable associations were identified among pediatric patients.

Most (58.0%) adult patients underwent a single surgical procedure during their hospital stay, but the total number of procedures per patient per hospital stay ranged from 1 to 5 (median = 1, IQR: 1, 2). The number of patients that underwent more than 1 (the median) surgical procedure increased over the study period. Although the number of surgeries performed annually increased over the decade-long study period (Table 4), there were no significant (*p* = .467) temporal trends in the risk of death over the period. The majority (54%) of patients underwent mitral valve replacement (Table 5). Additional procedures which were commonly performed included left atrial reduction (28.7%), double valve replacement (16.7%), and acquired tricuspid regurgitation repair (12.7%) (Table 5). The majority (61.3%) of surgeries performed on adult patients lasted less than four hours. Although duration of surgery was not significantly associated with death, it was a significant ( *p* = .016) predictor of sepsis among adults. Two surgical procedures had significant univariable associations with 30-day mortality: acquired tricuspid regurgitation repair (*p* = .011) and left atrial reduction (*p* = .026).

**Table 4:** Number of surgical procedures and deaths by year among adult and pediatric cardiac surgical patients seen at a tertiary hospital in Kenya, 2008–2017.

	**Adult Patients**				**Pediatric Patients**			
**Year**	**1 or 2 procedures**	**3 or more procedures**	**30-day Mortality**		**1 or 2 procedures**	**3 or more procedures**	**30-day Mortality**	
			**Frequency** **(x**^a^**/n**^b^**)**	**Percentage (%)**			**Frequency (x**^a^**/n**^b^**)**	**Percentage(%)**
2008	0	0	0/0	0	3	0	0/3	0
2009	4	0	0/4	0	0	0	0/0	0
2010	5	0	0/5	0	1	0	0/1	0
2011	4	0	1/4	25.0	0	0	0/0	0
2012	4	1	0/5	0	3	0	0/3	0
2013	9	3	1/12	8.3	1	0	0/1	0
2014	8	2	2/10	20.0	7	0	0/7	0
2015	2	2	0/4	0	3	0	1/3	33.3
2016	9	3	3/12	25.0	2	1	0/3	0
2017	69	25	8/94	8.5	10	0	1/10	10.0

**Notes.**

a Number of 30-day post-surgical deaths.

b Number of cardiac surgical patients.

**Table 5:** Selected surgical procedures and their unadjusted (univariable) associations with inhospital mortality and mediastinal hemorrhage among adult cardiac surgical patients seen at a tertiary hospital in Kenya, 2008–2017.

**Procedure**	**% Adult patients** **(*n* = 150)**	**In-Hospital mortality** *p*-value^a^	**Mediastinal hemorrhage** *p*-value^a^
Acquired tricuspid regurgitation repair	12.7	**.006**	.091
Appendectomy	15.3	.506	.313
Atrial septal defect closure	6.7	.940	.142
Aortic valve replacement	6.0	.850	.355
Aortic valve repair	0.7	.742	.505
Bentall’s procedure	2.7	.515	.395
Coronary artery bypass graft –1-2 grafts	10.0	.**015**	.813
Coronary artery bypass graft –3-4 grafts	4.7	.385	.902
Double valve replacement	16.7	.802	.751
Left atrial reduction	28.7	.064	.642
Left atrial thrombectomy	7.3	.977	.107
Maze procedure	7.3	.977	.269
Mitral cleft repair	1.3	.648	.344
Mitral valve replacement	54.0	.804	.955
Re-routing pulmonary veins to Left Atrium	0.7	.748	.505
Ventricular septal defect closure	0.7	.748	.505

**Notes.**

a Based on Chi-square test.

As was the case with adult patients, the majority (64.5%; median =1, IQR: 1, 2) of pediatric patients underwent a single procedure. Only one of the pediatric patients had more than three procedures during their hospital stay (Table 4). As with adult patients, the pediatric patients most often had mitral valve replacement (25.8%). Among pediatric patients, significant univariable associations were noted between double valve replacement and death (*p* = .046), left atrial reduction and death (*p* = .046), and left atrial reduction and mediastinal hemorrhage (*p* = .022). Duration of surgery was less than four hours for 64.5% of these patients. Surgical duration greater than four hours had significant univariable association with both development of sepsis (*p* = .049) and death (*p* = .049) among the pediatric patients.

The majority of both pediatric patients (96.8%) and adult patients (93.3%) underwent cardiopulmonary bypass (CPB). Neither the use of CPB nor the temperature range on CPB were significantly (*p* > .05) associated with overall 30-day mortality or in-hospital 30-day mortality for either age category. However, among patients who underwent any coronary artery bypass graft (CABG) procedure, all of whom were adults, temperature on CPB below normothermia had significant unadjusted association with 30-day mortality (*p* < .001). Use of cardioplegia was also common among both pediatric patients (93.5%) and adults (89.3%). However, neither use of cardioplegia nor duration of its use (<30 min vs ≥30 min), were significantly associated with 30-day mortality in either group. The above classification of duration of use of cardioplegia is in accordance with the standard dosing interval utilized at the hospital. There were no significant associations between any post-surgical complications and CPB use, CPB temperature, cardioplegia use, or duration of cardioplegia among pediatric patients.

Among adults, the use of CPB, as well as the use of cardioplegia had significant univariable associations with a limited number of other post-surgical complications, including acute kidney injury, pneumonia, and renal impairment (Table 6). Post-surgical renal status was classified as a functional grouping (normal versus all other categories of impairment); this had significant univariable associations with both CPB (*p* = .023) and use of cardioplegia (*p* = .002).

**Table 6:** Selected post-surgical outcomes and their unadjusted (univariable) associations with cardiopulmonary bypass and use of cardioplegia among adult cardiac surgical patients seen at a tertiary hospital in Kenya, 2008–2017.

**Outcomes**	**% CPB**^a^**Recipients** **(*n* = 140)**	*p*-value^b^	**% Cardioplegia Recipients** **(*n* = 134)**	*p*-value^b^
**Complications**				
Acute Kidney Injury	7.1	**<.001**	6.7	**<.001**
Atrial Fibrillation (Post-Surgical Onset)	14.3	.221	14.9	.215
Cardiogenic Shock	3.6	.543	3.7	.432
Days in Intensive Care Unit (>2)	63.6	.548	62.7	.316
Overall 30-day Mortality	10.0	1.0.	10.4	.597
Intra-surgical Mortality	2.1	.605	2.2	.605
In-hospital Post-operative Mortality	9.3	.940	9.7	.654
Mediastinal Hemorrhage	30.7	.962	29.9	.531
Pericardial Effusion	15.0	.187	14.9	.345
Pneumonia	5.0	.054	5.2	.247
Renal Impairment (Renal Status ≠ Normal)	26.4	**.023**	24.6	**.002**
Re-operated sternotomy	2.1	.640	2.2	.545
Re-operation (non-sternotomy)	0.7	.789	0.7	.729
**Positive outcomes**				
Days on Cardiac Ward (<4)	56.4	**.017**	56.7	**.022**
Resolved Diagnosis of Atrial Fibrillation	9.3	.221	9.0	.215
Resolved Diagnosis of Heart Failure	82.1	.341	82.1	.492

**Notes.**

a Cardiopulmonary bypass.

b Based on Chi-square test.

Positive surgical outcomes were also assessed for association with CPB and cardioplegia. Among adult patients, fewer than 4 days required on the cardiac ward following surgery had significant univariable associations with use of CPB (*p* = .017) and cardioplegia (*p* = .022) (Table 6).

### Predictors of surgical complications

Since the total number of pediatric patients was quite low, no multivariable models were built for pediatric patients. Logistic regression models, which included only adult patients, were fit separately for each severe complication as an outcome variable and adjusted for rare events using Firth bias corrected logistic regression model (Firth, 1993). Several models identified one or more predictors for a complication; however, most of the predictors were unique to each model and were not common across complication models (Table 7). The only common predictors of severe complications were age as a continuous variable (significant predictor in three models) and atrial fibrillation (significant in two models) (Table 7). The complications models for which Hosmer-Lemeshow results indicated good fit included models for overall 30-day mortality, acute kidney injury, pneumonia, and cardiogenic shock. Significant predictors of 30-day mortality were age in years (OR = 1.05, *p* = .015) and atrial fibrillation (OR = 4.12, *p* = .018). Similarly, significant predictors of acute kidney injury (AKI) were atrial fibrillation (OR = 18.25, *p* = .001), mitral valve replacement (OR = 0.14, *p* = .019), and use of cardiopulmonary bypass (CPB) during surgery (OR = 0.06, *p* = .002). The single significant predictor of in-hospital mortality was acquired tricuspid regurgitation repair (OR = 4.89, *p* = .010), while significant predictors of pneumonia were lower respiratory tract infection (OR = 10.50, *p* = .017) and age (OR = 1.06, *p* = .015). Finally, significant predictors of cardiogenic shock were thyroid derangement (OR = 15.35, *p* = .026) and age (OR = 1.07, *p* = .023).

**Table 7:** Results of final models showing predictors of pre- and peri-surgical of complications among adult cardiac surgical patients seen at a tertiary hospital in Kenya, 2008–2017.

**Outcome**	**Predictor**	**Odds ratio**	**95% CI**^a^**Lower limit**	**95% CI**^a^**Upper limit**	*p*-value^b^	**H-L**^c^ *p*-value
Overall 30-day mortality	Age in years	1.05	1.01	1.09	.015	.719
	Atrial fibrillation (Yes vs. No)	4.12	1.27	13.35	.018	
Acute kidney injury	Atrial fibrillation (Yes vs. No)	18.25	3.10	107.58	.001	.874
	Mitral valve replacement (Yes vs. No)	0.14	.0.03	0.72	.019	
	On cardiopulmonary bypass during surgery (Yes vs. No)	0.06	0.01	0.33	.002	
In-hospital mortality	Acquired tricuspid regurgitation repair (Yes vs. No)	4.89	1.47	16.33	.010	.
Pneumonia	Lower respiratory tract infection (Yes vs. No)	10.50	1.52	72.54	.017	.282
	Age in years	1.06	1.01	1.11	.015	
Cardiogenic shock	Thyroid derangement (Yes vs. No)	15.35	1.39	169.79	.026	.508
	Age in years	1.07	1.01	1.14	.023	
Sepsis	Duration of procedure (≥4 h vs. <4 h)	2.80	1.17	6.69	.021	
Stroke	Human immunodeficiency virus infection (Yes vs. No)	31.89	2.39	424.65	.009	

**Notes.**

a Confidence Interval.

b Based on Wald test.

c Hosmer-Lemeshow Goodness-of-fit test.

## Discussion

The objectives of this study were to describe the characteristics of cardiac surgical patients as well as identify the associations between these characteristics and morbidity and mortality events following cardiac surgery at a tertiary hospital in Kenya. Reducing complications among high-risk patients is an important goal for resource-limited settings. However, few studies have investigated the underlying patient characteristics or surgical decisions which may impact the development of complications in such settings. Identification of predictors of surgical outcomes is important in guiding clinical/surgical decisions to help reduce morbidity and mortality.

The high proportion of aortic and mitral valve defects in both adult and pediatric patients in this study is consistent with the high prevalence of rheumatic heart disease in sub-Saharan Africa (Damasceno et al., 2012; Bigna et al., 2017). Similarly, the adult patients in the current study had a high proportion (27%) of multiple valve involvement, providing further support for the potential role of advanced rheumatic heart disease as a contributor to the cardiac defects. Such patients require more aggressive intervention and often experience more severe post-surgical complications (Woldu & Bloomfield, 2016). Unfortunately, information on history of rheumatic fever was not available in the study data and, therefore, this could not be specifically investigated.

The predictors of complications identified in this study broadly coincide with those identified by the African Surgical Outcomes Study (ASOS) Group (Kluyts et al., 2018). Age is a prominent factor in complications, as is underlying quality of health. However, more detailed comparisons between predictors assessed in the current study and those of ASOS is not possible given the divergent goals of the studies. While the present study sought to identify predictors of complications of cardiac patients seen in a single tertiary health facility so as to guide local service improvement, the ASOS was designed to create a simple instrument to predict patient risk continent-wide with the goal of generalized risk reduction. The ASOS provides a general tool but largely reduces the risk calculations to limited demographic factors and type of surgery.

The current study developed a number of models which highlight specific and clinically useful associations between patient characteristics or procedures and morbidity and mortality among patients. Patient characteristics, particularly age and co-morbidities, played the greatest roles in predicting significant post-surgical complications. Age was a predictor of post-surgical complications in three of the seven final models developed and existing health conditions in five of the final models. Surgical factors were significant predictors in only three of the final models. Beyond the contribution of age, the co-morbidities identified by the final logistic regression models varied by specific complication. The variation in outcome prediction by co-morbidity points to the importance of careful assessment of underlying health issues as a means of improving outcomes for cardiac surgical patients.

Significant predictors of overall 30-day mortality were age (OR = 1.05) and atrial fibrillation (OR = 4.12). For the in-hospital death model, the odds of dying following surgery but before discharge was higher among patients undergoing acquired tricuspid regurgitation repair (OR = 4.89) than those that did not undergo this procedure. Other studies have pointed out the high mortality rate associated with this procedure with one study reporting a mortality rate of 10% (Fender, Zack & Nishimura, 2018). It is worth noting that all deaths in the present study occurred within 30 days post-surgery and were all due to cardiac causes. Hence, cause of death could not be investigated to assess potential associations between cause of death and pre-, peri-, and intra-surgical factors.

Although as many as 18% of cardiac surgical patients from resource-rich settings experience acute kidney injury (AKI) (Thiele, Isbell & Rosner, 2015), only 10.5% of the cardiac surgical patients in this study experienced the condition. This may be due to either differences in (a) definitions which have recently been expanded, or (b) the proportion of adult patients with pre-existing nephropathy or renal impairment. Despite the relatively low frequency of the complication in the present study, multivariable modeling identified pre-surgical diagnosis of atrial fibrillation as a risk factor (OR = 18.25) among adults, while mitral valve replacement (OR = 0.14) and the use of CPB during surgery (OR = 0.06) were identified as protective factors for acute kidney injury among adult patients. This is in contrast to expected associations, as use of CPB has been identified as a significant risk factor of AKI due to the mechanical and inflammatory insults of the intervention (Thiele, Isbell & Rosner, 2015). Although AKI has previously been significantly associated with hypertension (Ortega-Loubon et al., 2016) there was no significant association between the two in the final AKI model of the current study. Notwithstanding, careful assessment of pre- and post-operative patient use of anti-hypertensives would be a useful adjunct to future study of patient outcomes.

A univariable association was identified between temperature on CPB below normothermia and multiple outcomes including renal impairment, acute kidney injury and death among patients undergoing CABG procedures. While these findings are consistent with those of previous studies (Suleiman et al., 2011), temperature on CPB was not significant in any of the multivariable models. Despite the lack of associations, given the potential for minor changes such as adapting CPB temperature for different procedures or avoiding use of specific CPB-related products to produce meaningful reductions in mortality and development of other complications, additional investigations should be undertaken to identify CPB-related practices and associated complications.

The final pneumonia model included two significant predictors: lower respiratory tract infections (OR = 10.50) and age (OR = 1.06). The finding that the odds of pneumonia were higher among patients with lower respiratory tract infections is consistent with reports from previous studies (Carrel et al., 2001). Contemporaneous reports encourage judicious use of corticosteroids in the peri-operative period and increased scrutiny with regard to lung function both pre- and post-operatively to ensure post-surgical improvement (Topal & Eren, 2012). Age is a well-described risk factor for pneumonia and adverse outcomes associated with the diagnosis (Strobel et al., 2016). The odds ratio for age identified in the present study is similar to that identified by Kilic et al. in their report on development of a predictive risk model for pneumonia following cardiac surgery (Kilic et al., 2016).

In the present study, the final model for development of post-surgical cardiogenic shock included thyroid derangement (OR = 15.35) and age (OR = 1.07). This finding coincides with a longstanding body of evidence implicating thyroid dysfunction in the cascade of cardiac disease generally and specific studies demonstrating the role of T3 in low cardiac output among CABG patients (Cerillo et al., 2014). The final sepsis model assessed risk among adults and included a single predictor, duration of procedure >4 h (OR = 2.80). However, since the model did not fit the data well, the findings are not considered robust. Similarly, the final model for stroke development included the variable HIV as a significant predictor (OR = 31.89), but the model did not fit the data well. Although details regarding stroke were not captured in the data used in the present study, the predictive role is borne out by the association identified in prior studies (Benjamin et al., 2012). However, it is worth noting that the proportion of cardiac surgical patients with HIV in the current study was very low. Therefore, ongoing investigation of this association is recommended, especially as antiretroviral therapy uptake increases.

Substance use was anticipated to be a potential risk factor for post-surgical complications. However, univariable associations between various complications and smoking or alcohol use did not maintain their significance in the final adjusted models. Reported substance use did not conform to expected levels: whereas tobacco use was lower than the reported national proportion (9.3% vs. 13.5% of adults), alcohol use was far higher than that reported nationally (22.7% vs. 13.6% of adults) (National Authority for the Campaign Against Alcohol and Drug Abuse, 2012; Ngaruiya et al., 2018). It is worth noting, however, that the present study did not include data on amount/quantity of substance use, rendering the variable not very clinically meaningful. The ASOS surgical risk calculator reported similar findings with regard to smoking, and the factor was excluded from the final model of general risk assessment (Kluyts et al., 2018).

Challenges to initiating and maintaining a cardiac surgical program in a resource-limited setting are many. Over the study period, the hospital has addressed many of these challenges, with a particular focus on increasing planning and staff training (Muturi, 2008). The increasing capacity of the facility over time is evidenced by the trend toward greater numbers of procedures performed each year, with a large increase noted in 2017. Along with the increase in total surgeries performed, the proportion of hospitalizations which included three or more procedures has increased over time, which may indicate augmentation of staff capability or an increase in complex case referrals over the study period. The deaths in each year did not increase apace with the number of surgeries, as evidenced by the lack of significant temporal trend, which is consistent with the advances in staff experience and facility capacity. A recent case study highlighted the effectiveness of the so-called “Nigerian model” in which provision of ongoing training opportunities for local staff is prioritized, both through out-of-country training, where feasible or supported by government entities, as well as through acceptance of external aid and development of partnerships (Oludara et al., 2014). Continued assessment of surgical outcomes at the study hospital should likewise be undertaken to provide data-driven appraisal of both the need for additional training and the effectiveness of such training or other changes in resource allocation on patient outcomes.

### Strengths and limitations

The use of Firth logistic models in this study is an important strength. These models were used because the maximum likelihood estimation of the ordinary logistic model suffers from small-sample bias. In situations when the events are rare, Firth logistic models provide better estimates than the ordinary logistic models (Heinze & Schemper, 2002). The strength of the Firth model is that it uses penalized likelihood to reduce small-sample bias in maximum likelihood estimation (Firth, 1993). Use of penalized likelihood also has the strength of producing finite, consistent estimates of regression parameters in situations when maximum likelihood estimates do not exist due to complete or quasi-complete separation (Heinze, 2006; Williams, 2019). Thus, although small sample size was a statistical challenge, it was adequately addressed using appropriate statistical methodology. However, it should be pointed out that the small number of complication events in this study is a positive clinical outcome. Despite the sample size challenges, the present study provides useful descriptive information for clinicians in this hospital and may be useful for guiding future larger studies in other low resource settings. The findings suggest that patient characteristics may be more important contributors to the observed incidence of complications than surgical practices. However, these will need to be further confirmed in larger studies.

### Conclusion

The findings of this study indicate that patient characteristics and, most importantly, co-morbidities, may provide some clues regarding the likelihood of developing post-surgical complications, whereas intra-surgical factors play a much more circumscribed role. Future larger studies, involving more patients, will need to further investigate these to generate information that will be helpful for guiding clinical decisions.

##  Supplemental Information

10.7717/peerj.11191/supp-1 Supplemental Information 1 Data Dictionary Contains descriptions of the variables in the dataset.

10.7717/peerj.11191/supp-2 Supplemental Information 2 Project Dataset. De-identified dataset contaiining all variables used in the analysis

## Additional Information and Declarations

### Data Availability

The following information was supplied regarding data availability: The raw data and data dictionary are available in the Supplementary Files.
